# Epithelium-derived exosomes promote silica nanoparticles-induced pulmonary fibroblast activation and collagen deposition via modulating fibrotic signaling pathways and their epigenetic regulations

**DOI:** 10.1186/s12951-024-02609-y

**Published:** 2024-06-12

**Authors:** Yan Li, Hailin Xu, Ying Wang, Yurou Zhu, Kun Xu, Zhu Yang, Yanbo Li, Caixia Guo

**Affiliations:** 1 Department of Occupational Health and Environmental Health, School of Public Health, Capital Medical University, No.10 Xitoutiao, You An Men, Beijing, 100069 China; 2 Beijing Key Laboratory of Environmental Toxicology, Capital Medical University, No.10 Xitoutiao, You An Men, Beijing, 100069 China; 3 Department of Toxicology and Sanitary Chemistry, School of Public Health, Capital Medical University, No.10 Xitoutiao, You An Men, Beijing, 100069 China; 4 School of Medicine, Hunan Normal University, Changsha, 410013 Hunan China; 5 State Key Laboratory of Environmental and Biological Analysis, Hong Kong Baptist University, Hong Kong, China

**Keywords:** Silica nanoparticles, Pulmonary toxicity, Fibroblast activation, Epithelial-fibroblast communication, Exosomes, miR-494-3p

## Abstract

**Background:**

In the context of increasing exposure to silica nanoparticles (SiNPs) and ensuing respiratory health risks, emerging evidence has suggested that SiNPs can cause a series of pathological lung injuries, including fibrotic lesions. However, the underlying mediators in the lung fibrogenesis caused by SiNPs have not yet been elucidated.

**Results:**

The in vivo investigation verified that long-term inhalation exposure to SiNPs induced fibroblast activation and collagen deposition in the rat lungs*. *In vitro, the uptake of exosomes derived from SiNPs-stimulated lung epithelial cells (BEAS-2B) by fibroblasts (MRC-5) enhanced its proliferation, adhesion, and activation. In particular, the mechanistic investigation revealed SiNPs stimulated an increase of epithelium-secreted exosomal miR-494-3p and thereby disrupted the TGF-β/BMPR2/Smad pathway in fibroblasts via targeting bone morphogenetic protein receptor 2 (BMPR2), ultimately resulting in fibroblast activation and collagen deposition. Conversely, the inhibitor of exosomes, GW4869, can abolish the induction of upregulated miR-494-3p and fibroblast activation in MRC-5 cells by the SiNPs-treated supernatants of BEAS-2B. Besides, inhibiting miR-494-3p or overexpression of BMPR2 could ameliorate fibroblast activation by interfering with the TGF-β/BMPR2/Smad pathway.

**Conclusions:**

Our data suggested pulmonary epithelium-derived exosomes serve an essential role in fibroblast activation and collagen deposition in the lungs upon SiNPs stimuli, in particular, attributing to exosomal miR-494-3p targeting BMPR2 to modulate TGF-β/BMPR2/Smad pathway. Hence, strategies targeting exosomes could be a new avenue in developing therapeutics against lung injury elicited by SiNPs.

**Graphical Abstract:**

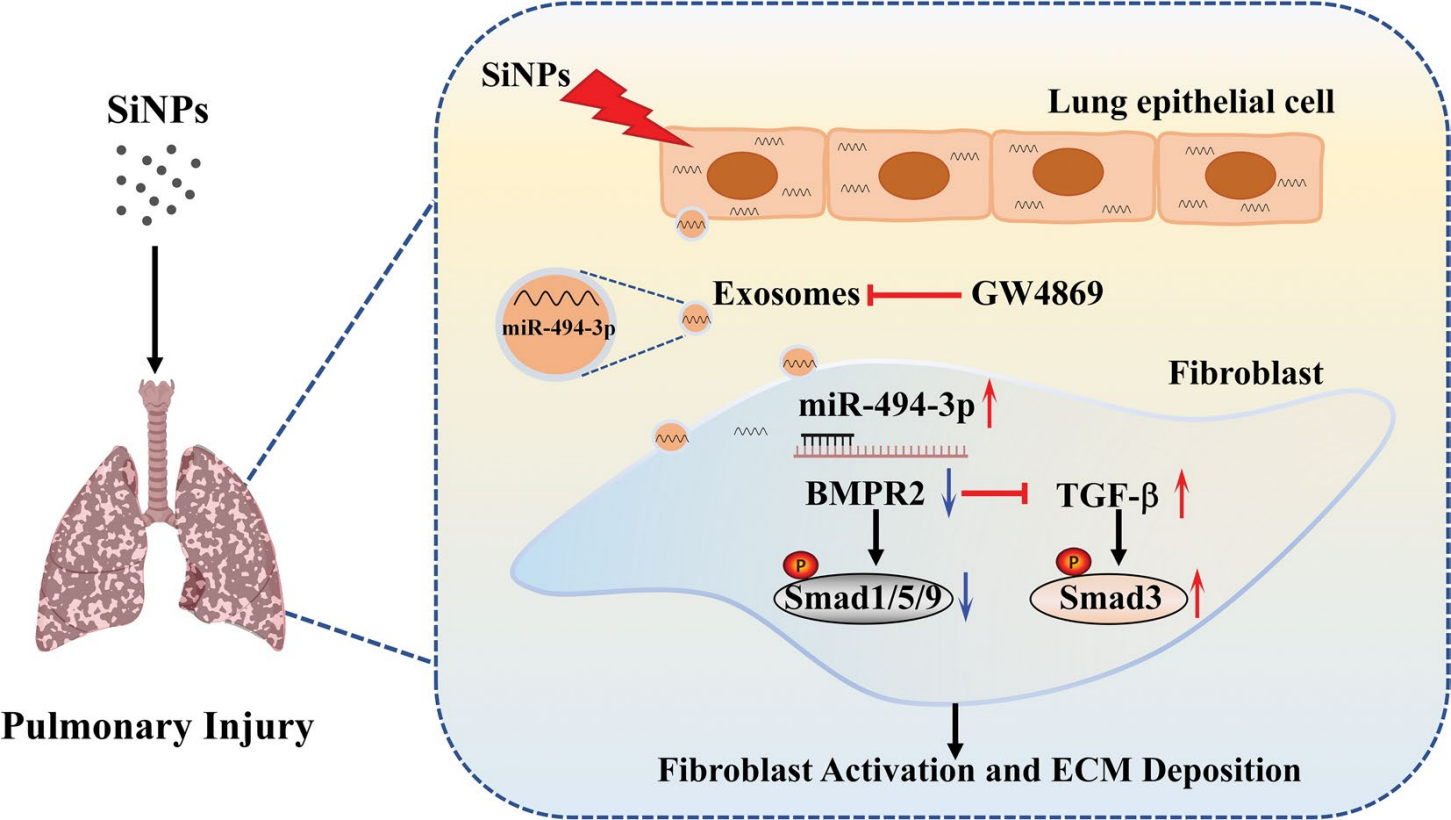

**Supplementary Information:**

The online version contains supplementary material available at 10.1186/s12951-024-02609-y.

## Introduction

The rapid advancement of nanotechnology has brought about an urgent need to assess the potential toxicological impacts of nanoparticles on both the environment and human health. Silica nanoparticles (SiNPs) are commonly used nanomaterials, finding their way into a wide range of products, food additives, and biomedical applications. Inhalation was recognized as the most significant route of exposure to SiNPs, particularly in the workplace. It has been shown that nanoparticles presented in welding fumes may contribute, at least in part, to pulmonary inflammation [1]. Liao et al. found pulmonary dysfunction in SiNPs handling workers, characterized by a decreased 25% forced expiratory flow (FEF25%) [2]. Increasing animal studies have shown that SiNPs could cause pulmonary oxidative stress, mitochondrial dysfunction, inflammation, DNA damage, and epigenetic alterations, eventually contributing to lung fibrotic lesions [3–6]. Nevertheless, the pathogenesis and underlying mediators remain incompletely understood, and therapeutic targets and effective treatments are lacking.

The respiratory epithelium serves as a first line of contact with harmful substances. Studies have demonstrated that epithelium injury and dysfunction by repeated and sequential inhaled stimuli promoted mesenchymal fibroblast activation [7], resulting in collagen deposition and lung fibrogenesis [8]. In particular, exosomes can serve as mediators for cell-to-cell communication through transferring information packages [9–11]. Exosomes with a lipid bilayer membrane structure are known as extracellular vesicles (EVs; 30–150 nm), formed by inward depression of the internal membrane during endocytosis and released into the extracellular process after fusion with the plasma membrane [12, 13]. Exosomes contain biomolecules [14], including lipids, proteins, and nucleic acids (miRNA, mRNA). Notably, most exosomal biologic effects have been ascribed to miRNAs through epigenetic regulation [15]. Emerging data have indicated that the miRNAs in exosomes (also named exosomal miRNA) act as a key mediator in regulating cell differentiation and phenotypic changes [16, 17]. For example, Njock et al*.* have illustrated that exosomal miRNAs can regulate the alteration of pulmonary function [18]. Previous studies have reported aberrant miRNA expression profiles in SiNPs-stimulated lung tissue and in vitro cultured lung cells [19–21], where miRNAs may participate in epithelial-fibroblast communication [22, 23]. Despite these advances, the specific role of exosomes/exosomal miRNA in the epithelial-fibroblast cross-talk and resultant pulmonary fibroblast activation and collagen deposition upon SiNPs exposure remains unclear.

Based on these findings, this work focused on elucidating the role and regulatory mechanism of exosomal miRNAs from lung epithelial cells in fibroblast activation and subsequential collagen deposition. The differential miRNAs in exosomes derived from SiNPs-stimulated lung epithelial cells were screened via transcriptome sequencing. Then, the regulatory mechanism for exosomal miRNAs on fibroblast activation and collagen proliferation was explored in vivo and in vitro.

## Methods

### Animal experiment and ethics statement

The chronic inhalation exposure model of SiNPs was established in Wistar rats through intratracheal instillation. In brief, male Wistar rats (6-w old) were acquired from the Experimental Animal Center of Capital Medical University (Beijing, China) and lived in comfortable surroundings with plenty of water and food, maintained a constant humidity of 50 ± 5%, specific temperature of 24 ± 1 °C under 12-h light/dark cycles. After 1-w adaptation, experimental animals were randomly assigned to four groups, including one control group and three SiNPs exposed groups. In the exposed groups, rats were administered with SiNPs suspension via intratracheal instillation at doses of 1.5, 3.0, and 6.0 mg/kg·bw, respectively, once a week for a total of 24 treatments. As previously described [24], the SiNPs used in this experiment were synthesized using the Stöber approach, and their detailed characterization was described in the additional file Fig. S1 and Table S1. Its applied dosage was based on previous inhalation studies of SiNPs [25, 26], where the administered dose was evaluated based on occupational exposure scenarios. The rats were intratracheally instilled with 0.9% saline in the control group. Finally, the rats fasted overnight, and tissues were harvested and stored at − 80 °C. The animal experiment was carried out following the Animal Care and Use Committee at Capital Medical University (Ethical number: AEEI-2021-269).

### Histopathology

Fresh rat lung tissues were placed in a fixative solution and embedded in paraffin. Subsequently, hematoxylin–eosin (HE; Servicebio, China) and Masson staining (Servicebio, China) were operated on to evaluate histopathological damage following the protocol. The stained slides were scanned with a Pannoramic automated slide scanner (3DHISTECH, Hungary). Masson staining was used to detect the degree of collagen deposition in rat lungs, and its corresponding semi-quantification was conducted using Image J software. The ratio of the blue-positive stained area to all of the lung interstitial area was calculated, known as collagen volume fraction (CVF). The severity of pulmonary injury was estimated using the Ashcroft Score, as previously described [27].

### Hydroxyproline content detection

Hydroxyproline (HYP) is known as the sensitive biochemical marker that indicates collagen fiber changes [28] and the severity of fibrosis [29]. In animal tissues, HYP is essentially found in collagen [30]. The HYP measurement was recommended as the “gold standard” for preclinical evaluation of pulmonary fibrosis by the 2017 American Thoracic Society guidelines [31]. According to the product’s manual, the hydroxyproline (HYP) content in lungs (50 mg) or culture supernatants was determined through a Hydroxyproline Assay Kit (Jiancheng, China).

### Cell culture and exposure

Human lung epithelial cells (BEAS-2B cells) were obtained from Cell Resource Center (Shanghai, China) and seeded in DMEM medium (Corning, USA) containing 10% fetal bovine serum (FBS; Gibco, Australia) within a cell incubator (Thermo Fisher Scientific, USA). The BEAS-2B cells were incubated until 80% confluency and then treated with SiNPs (0 or 25 μg/mL) for 24 h. To investigate molecular mechanisms, GW4869 was applied to assess the impact of exosomes derived from BEAS-2B cells on fibroblast activation. GW4869, as a potent, specific, non-competitive inhibitor of membrane-neutral sphingomyelinase, is known to reduce exosome release significantly [32] and barely has toxicity to cultured cells [33]. BEAS-2B cells were preprocessed with GW4869 (5 μM; Selleck, USA) for 24 h [34] and then subjected to 25 μg/mL SiNPs for another 24 h. Given that GW4869 is insoluble in water but soluble in DMSO, we set a solvent control treated with an equal amount of DMSO. Lastly, the BEAS-2B cell supernatants (SB) were gathered and filtered with filter membranes (0.22 μm; Millipore, Billerica, USA).

Human lung fibroblast cells (MRC-5) were acquired from the Cell Resource of the Chinese Academy of Science (Beijing, China) and seeded in MEM medium (HyClone, USA) containing 10% FBS (Gibco, Australia) and 1% Non-Essential Amino Acids (NEAA; Gibco, USA) in a cell incubator. To investigate the epithelium-fibroblast communication, MRC-5 cells in the logarithmic phase with about 80% confluence were stimulated by the culture supernatants from BEAS-2B cells without or with SiNPs (named as SB-Ctr or SB-SiNPs).

### Cell proliferation assay

According to the protocol, fibroblast proliferation was assessed by cell counting-8 (CCK-8; Dojindo Laboratories, Japan). Briefly, the relative cell viability was expressed with 100% control cell viability, based on OD at 450 nm in the microplate reader (BioTek, USA).

### Wound healing assay

When the cultured MRC-5 cells in a 6-well plate were grown to 100% confluency, fibroblasts were scratched with a sterile 200 μL pipette tip and recorded as 0 h scratch status under the microscope (Leica, Germany). Afterward, cells were cultured in SB-Ctr or SB-SiNPs for 24 h and again examined microscopically (Leica, Germany). Image J software analyzed the wound healing rate using the mathematical equation: wound healing rate (%) = (0 h scratch area—24 h scratch area)/0 h scratch area × 100%.

### Real-time cell analysis (RTCA)

MRC-5 cells were cultured in RTCA E-plates (ACEA Biosciences; Germany) for 24 h and then incubated with the SB-Ctr or SB-SiNPs for another 24 h by changing half of the medium. The cell growth status was monitored in real-time, and the signals were collected every 15 min until 24 h. The analysis was performed using the xCELLigence RTCA instrument (ACEA Biosciences, Germany).

### Immunofluorescence

MRC-5 cells were placed in 4% paraformaldehyde, permeabilizated in 0.3% Triton X-100, blocked in 3% BSA, and incubated with α-smooth muscle actin (α-SMA) antibody (4℃, overnight; Abcam, USA), followed by incubation with fluorescence-labeled secondary antibody (room temperature, 1 h; anti-rabbit IgG; Abcam, USA). Lastly, the cell images were taken by laser scanning confocal microscopy (LSCM; Ti-2, Nikon, Japan).

### Exosomes extraction and characterization

The exosomes derived from BEAS-2B cells, including SB-Ctr or SB-SiNPs, were extracted following previous publishing [10, 35, 36]. In brief, the supernatants were centrifuged (2,000 g; 10 min; 4 °C) and filtered to discard cellular debris and microvesicles. Then, the supernatants underwent serial centrifugation at 10,000*g* for 30 min and 100,000*g* for 70 min using an SW32Ti rotor (Beckman, USA) at 4 °C. The pellets were further purified by being dissolved in phosphate buffered solution (PBS; Servicebio, China) and ultracentrifuged at 100,000*g* for 70 min at 4 °C. Lastly, the exosomes were resuspended with PBS.

Protein concentrations of exosomes were measured by the BCA Protein Assay Kit (Dingguo, China). Exosome morphology was reviewed by transmission electron microscopy (TEM; JEM2100, Japan). The concentration and size of exosomes were measured by ZetaVIEW S/N 17-310 instrument (Particle Metrix, Germany). Western blot was applied to identify exosome-specific protein markers, including CD9, CD63, TSG101, and calnexin [37].

### Exosomes uptake assay

The MRC-5 cells were incubated in confocal dishes to 80% confluence and cultured with DIO-labeled exosomes for 24 h. Nuclei were stained with Hoechst 33342 (10 μM; ThermoFisher Scientific, USA) for 30 min. LSCM examined the uptake of exosomes by MRC-5 cells.

### RNA isolation and RT-qPCR analysis

The total RNA in the exosomes, cells, and lung tissues was isolated using a Trizol reagent (Life Technologies, USA) following the product manual. Based on RNA quantification spectrophotometrically at 260 nm and 280 nm, the RNA (1 μg) was reverse transcribed into cDNA for mRNA and miRNA detection through PrimeScript™ RT reagent Kit with gDNA Eraser (Takara, Japan) and Mir-X ™ miRNA First-Strand Synthesis Kit (Takara, Japan), respectively. The level of miR-494-3p and mRNA expressions of collagen type I alpha 1 chain (*COL1A1*), collagen type III alpha 1 chain (*COL3A1*), fibronectin 1 (*FN1*), *α-SMA* and bone morphogenetic protein receptor 2 (*BMPR2*) were detected by TB Green® qRT-PCR kit (Takara, Japan). Data were normalized to *ACTB* (mRNA) or U6 (miRNA). The primer sequences involved are presented in Table S2.

### miRNA array analysis and luciferase reporter assay

Total RNA was extracted from exosomes, and miRNA microarray analysis was conducted using Agilent Arrays (Biotechnology, China). The differentially expressed genes were served as down- or upregulated with *p* < 0.05 and the absolute fold change > 1.5. Then, the target gene prediction was applied using TargetScan, miRDB, miRWalk, and StarBase databases. According to the bioinformatics analysis, the binding site between bone morphogenetic protein receptor 2 (*BMPR2)* and miR-494-3p was affirmed by the Dual-Luciferase Reporter Assay System (Promega, USA). In brief, the wild and mutant reporter plasmids of BMPR2-3 'UTR were constructed with pmirGLO vector and transfected into 293T cells. Lastly, bioluminescence was read at 48 h post-transfection.

### RNA interference

The anti-miR-494-3p (Oligobio, China) was transfected into BEAS-2B cells with RNA TransMate (Sangon Biotech, China) for 12 h before 24-h treatment with SiNPs (25 μg/mL). The groups were anti-miR-NC (negative control), anti-miR-494-3p, anti-miR-NC combined with SiNPs, and anti-miR-494-3p combined with SiNPs. Then, the culture supernatants from BEAS-2B cells were collected, filtered, and treated to MRC-5 cells to evaluate the impact of lung epithelial cell-derived miR-494-3p on fibroblast activation.

### Overexpression of BMPR2 by lentiviral transfection

BMPR2 overexpression lentivirus (OE-BMPR2) and negative control lentivirus (OE-NC) were purchased from Oligobio. MRC-5 cells were routinely seeded in six-well plates for 24 h. Subsequently, the culture medium was substituted with serum-free medium (1 mL), and the MRC-5 cells were transfected with OE-BMPR2 or OE-NC lentivirus (Oligobio, China) at a multiplicity of infection (MOI) of 10 along with the 8 μg/mL polybrene (Oligobio, China) for 24 h. Afterward, the culture medium was changed to a complete medium for another 48 h. To note, GFP expression of the lentivirus reporter gene was viewed, and cells exhibiting a fluorescence rate of at least 80% were applied in the following experiments.

### Western blot

The protein expression was tested by Western blot analysis and normalized by GAPDH. In brief, the total proteins from cultured cells, lung tissue, or exosomes were abstracted using a Protein Extraction kit (KeyGEN, China) and then quantified with BCA assay (Dingguo, China). The samples (20 μg) were subjected to sodium dodecyl sulfate–polyacrylamide (SDS-PAGE) gels and then blotted on polyvinylidene difluoride (PVDF) membranes, subsequently blocked with 5% milk (room temperature, 2 h). After three times washing with Tris-buffered saline with 0.05% Tween 20, the membrane was incubated with primary (4 °C, overnight) and secondary antibodies (room temperature, 2 h) diluents. The primary antibodies for CD9, CD63, BMPR2, Smad1, transforming growth factor beta (TGF-β), COL3A1 and FN1 were obtained from Santa Cruz, USA, and that for TSG101, calnexin, α-SMA from Abcam, UK, and that for *p*-Smad1/5/9, Smad3, *p*-Smad3, COL1A1 and GAPDH from CST, USA. The anti-mouse or -rabbit IgG was acquired from CST, USA. At last, the images were captured using the enhanced chemiluminescence (ECL) method and analyzed using Image J software.

### Statistical analysis

All statistical differences were conducted through SPSS 22.0 software. Data were expressed as mean ± standard deviation (SD). Student's t-test was used to compare two groups and ANOVA to multiple comparisons. A two-tailed Pearson correlation test was applied to describe the correlation between miR-494-3p and *BMPR2* levels. *p* < 0.05 manifests statistical significance.

## Results

### SiNPs caused fibroblast activation and collagen deposition in vivo

**Fig. 1:**
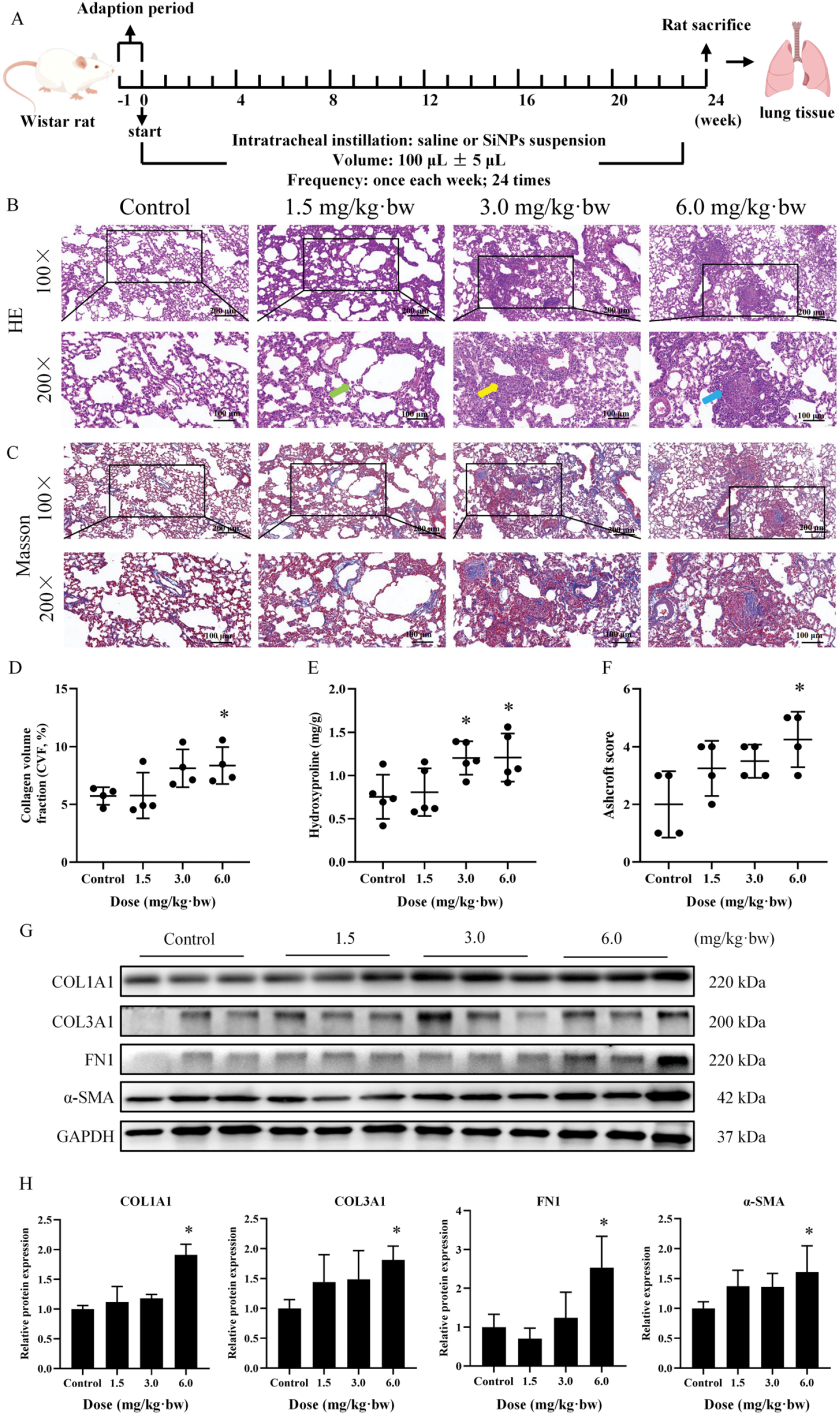
SiNPs promoted fibroblast activation and collagen deposition in vivo. **A** Schematic representation of animal experimental procedure. **B** HE staining. Alveolar structural destruction (green arrow), thickened alveolar walls (yellow arrow), and cell nodules (blue arrow) were observed in the lungs with SiNPs administration. N = 4. Representative images of Masson staining **C** and corresponding semi-quantitative result **D** illustrated a dose-dependent increase in collagen deposition. N = 4. Scale bar, 100 or 200 μm. **E** HYP measurement in the lungs. N = 5. (**F**) Ashcroft score was used to assess the severity of pulmonary fibrosis. N = 4. **G**, **H** Western blot assay to detect expressions of fibroblast activation and ECM deposition-associated markers, such as COL1A1, COL3A1, FN1, and α-SMA. N = 3. *p < 0.05 *vs* control

Firstly, the impact of SiNPs on lungs was assessed by establishing an in vivo model, and the specific experimental details were shown in Fig. 1A. HE staining of lung sections (Fig. 1B) indicated the alveolar structure was basically normal, without obvious destruction, and inflammatory infiltration in the control group. On the contrary, significant lung injuries were presented in SiNPs-exposed rats, as featured by widened alveolar septa, consolidation of alveolar cavities, and even cell nodules. Masson staining and its semi-quantitative (Fig. 1C, D) revealed that SiNPs promoted collagen accumulation in the rat lungs. Concertedly, the HYP content in the rat lungs (Fig. 1E) and Ashcroft score analysis (Fig. 1F) revealed that SiNPs induced pulmonary impairments with dose-dependent exacerbation, leading to lung fibrotic phenotypic changes. Simultaneously, up-regulated protein expressions, including COL1A1, COL3A1, FN1, and α-SMA, were detected in SiNPs-exposed rat lungs (Fig. 1G, H), indicating fibroblast activation and excessive accumulation of extracellular matrix (ECM) to contribute to pulmonary fibroplasia. Taken together, these data revealed that respiratory exposure to SiNPs via intratracheal instillation caused fibroblast activation, collagen, and ECM deposition, and the resultant lung fibrotic lesion.

### SiNPs-stimulated supernatants from pulmonary epithelial cells triggered fibroblast proliferation, migration, activation, and collagen deposition in vitro

**Fig. 2:**
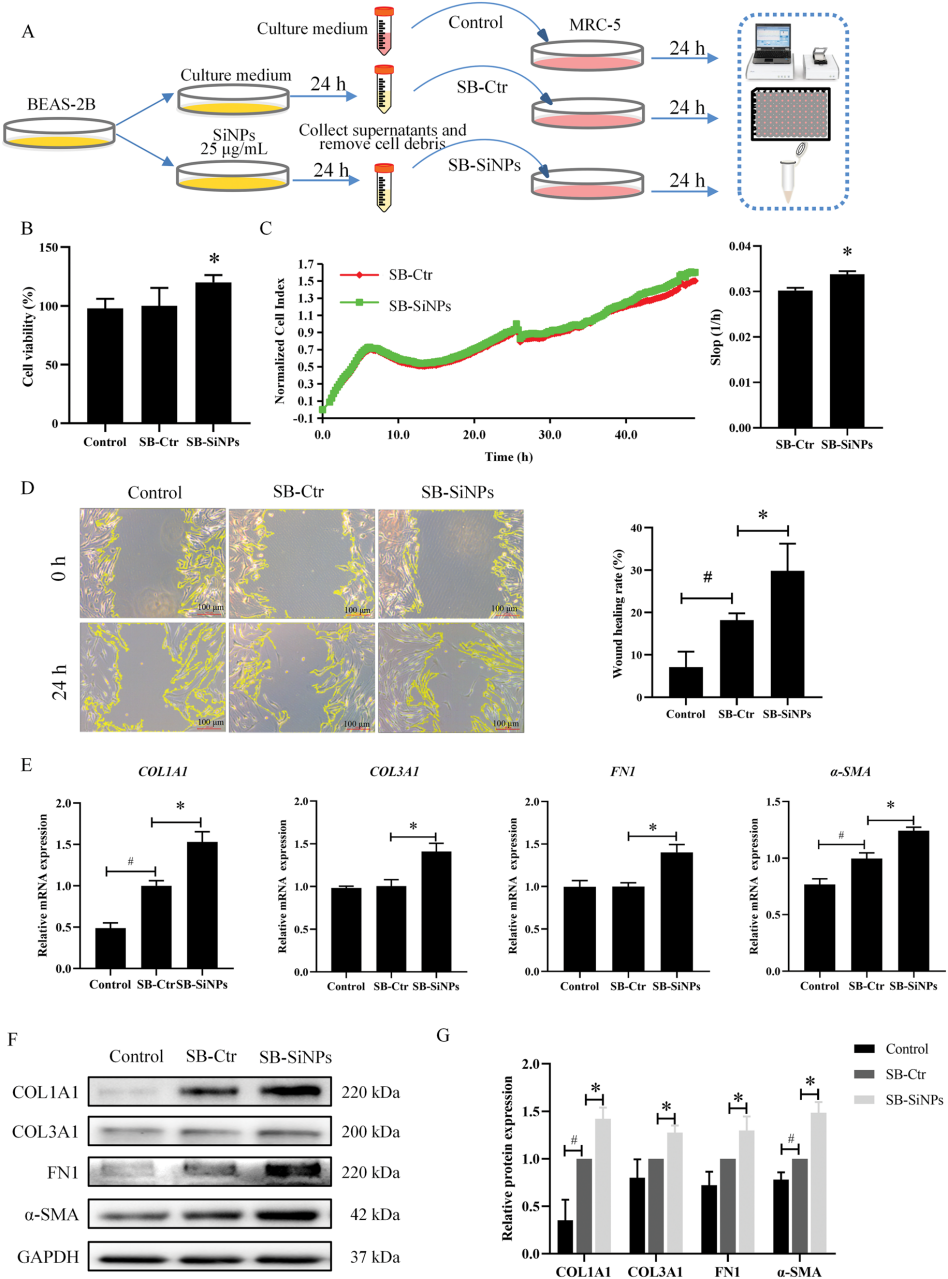
The supernatants from SiNPs-stimulated lung epithelial cells triggered fibroblast activation and collagen deposition in vitro. **A** Schematic representation of experimental procedure in vitro by which MRC-5 cells were stimulated by supernatants of BEAS-2B. **B** The cell proliferation of MRC-5 cells was analyzed using a CCK-8 assay. RTCA was also applied for the proliferation assessment of MRC-5 cells (**C**). Then, the migration of MRC-5 was detected by scratch assay (**D**). Scale bar, 100 μm. The expressions of fibroblast activation and ECM deposition-related regulators (including COL1A1, COL3A1, FN1, and α-SMA) were analyzed by RT-qPCR (**E)** and Western blot (**F**, **G**). N = 3; *p < 0.05 *vs* SB-Ctr; ^#^*p* < 0.05 *vs* control. ***SB-Ctr***, supernatants of normal BEAS-2B cells; ***SB-SiNPs***, supernatants of SiNPs-exposed (25 μg/mL) BEAS-2B cells

Given the pathogenesis of pulmonary fibroplasia as the result of repeated and sequential inhaled stimuli-induced injury to lung epithelium [38, 39], we next evaluated the intercommunication between epithelial cells and fibroblasts in response to SiNPs stimulation by establishing an indirect co-culture system in vitro. The specific experimental details are shown in Fig. 2A. Based on the cell viability determination in our previous publishing [40], here, the administered dosage of SiNPs to BEAS-2B cells was set to be 25 μg/mL, where cell viability remained up to 75%. The effects of supernatants secreted by BEAS-2B cells on MRC-5 cell proliferation were investigated via CCK-8 assay and RTCA. In comparison to *SB-Ctr*, SB-SiNPs exposure for 24 h resulted in a prominent rise in cell viability of MRC-5 (Fig. 2B). The enhanced fibroblast proliferation upon SB-SiNPs stimuli was also confirmed by the RTCA system (Fig. 2C). In addition, the wound healing assay demonstrated a significantly enhanced cell migration ability of MRC-5 cells when treated with SB-SiNPs (Fig. 2D). To evaluate the status of fibroblast activation and the deposition of collagen and ECM, we measured the mRNA and protein levels of COL1A1, COL3A1, α-SMA and FN1 (Fig. 2E–G). In the SB-SiNPs group, higher expressions of COL1A1, COL3A1, α-SMA, and FN1 in MRC-5 cells were noticed compared to that in the SB-Ctr group. These data confirmed the induction of fibroblast proliferation, migration, and activation in the context of lung epithelial injury elicited by SiNPs.

### Exosomes from lung epithelial cells as a key mediator for fibroblast activation upon SiNPs exposure

**Fig. 3:**
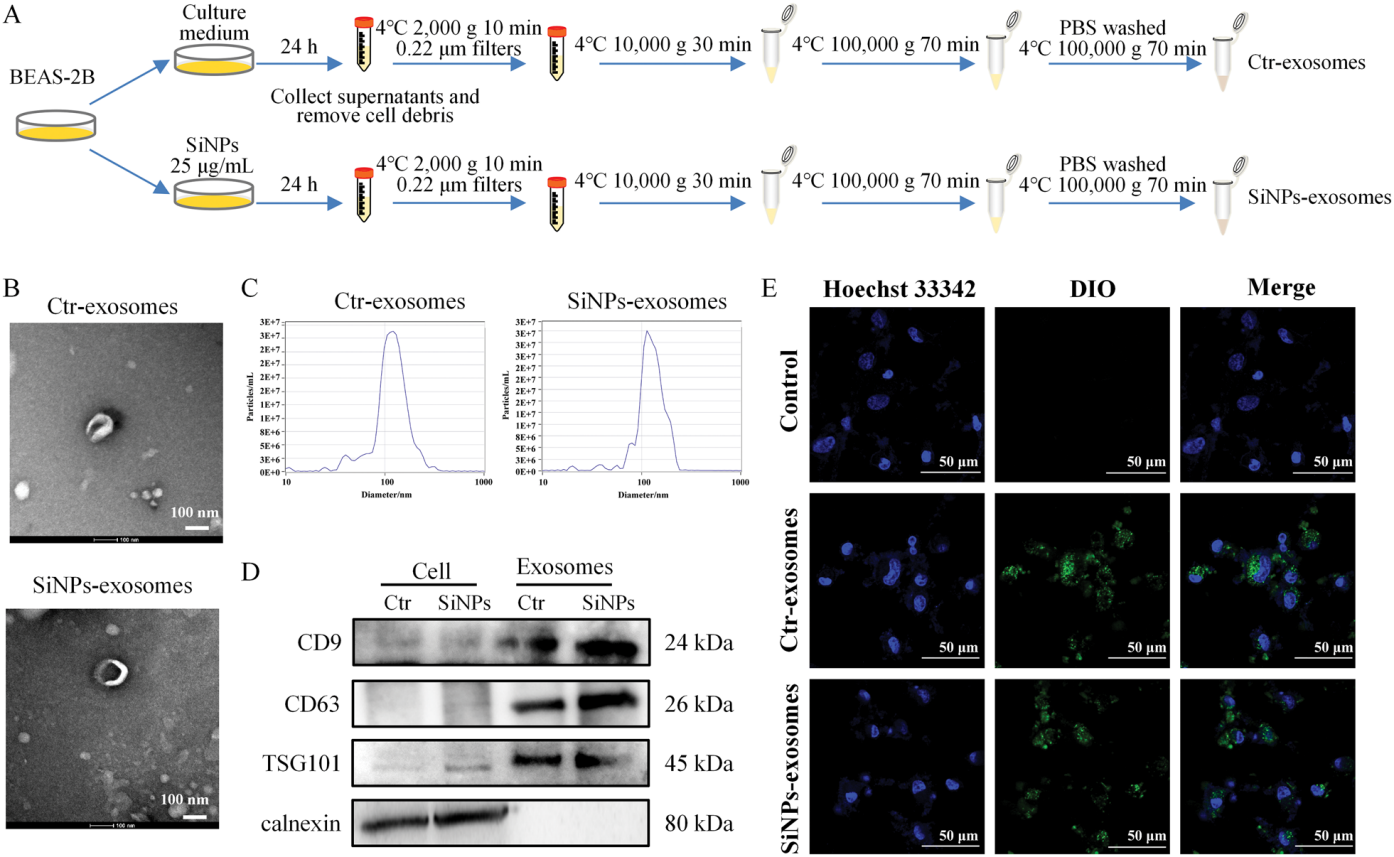
BEAS-2B-derived exosomes could be transported to MRC-5 cells. **A** Experimental procedures for exosome extraction. **B** Typical TEM images of exosomes. Scale bar, 100 nm. **C** The size of exosomes was detected. **D** The expressions of exosome markers, including CD9, CD63, TSG101, and calnexin, were assessed by Western blot. **E** Representative LSCM images presented that BEAS-2B cells-derived exosomes could be uptake by MRC-5 cells. Green: DIO-labeled exosomes; Blue: Hoechst 33342. Scale bar, 50 μm

**Fig. 4:**
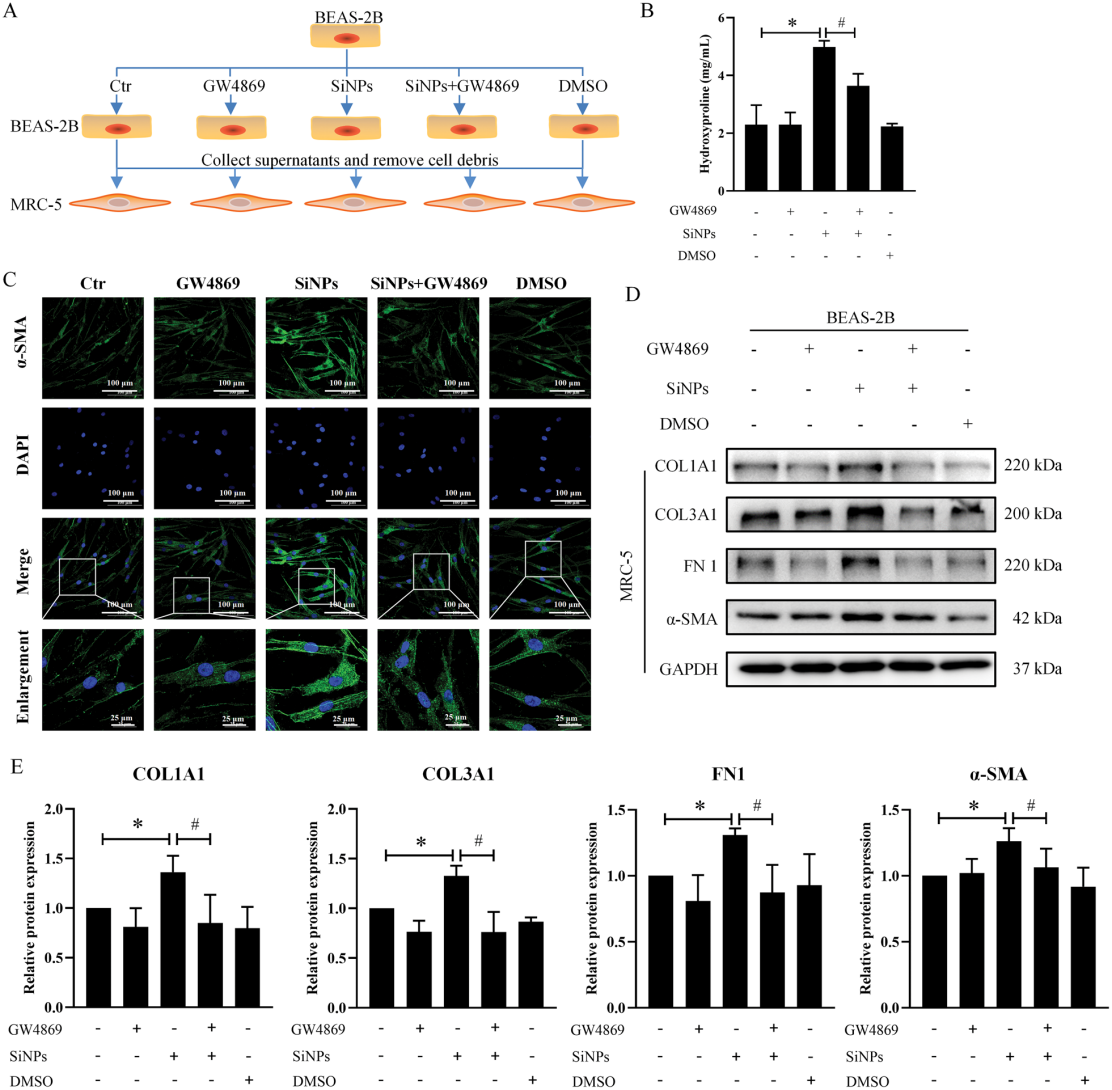
GW4869 attenuated SiNPs-induced fibroblast activation and collagen deposition. **A** The schematic diagram of cell treatment and grouping. BEAS-2B cells were preprocessed with GW4869 for 24 h and then treated with 25 μg/mL SiNPs for another 24 h. After treatment, the supernatants from BEAS-2B were collected and stimulated to MRC-5 cells for the evaluation of fibroblast activation. **B** The content of HYP was measured in supernatants of MRC-5. **C** Immunofluorescence staining for α-SMA in MRC-5 cells. Green: α-SMA; Blue: DAPI. Scale bar, 25 or 100 μm. Meanwhile, protein levels of COL1A1, COL3A1, FN1, and α-SMA were quantified via Western blot in MRC-5 cells (**D**, **E**). N = 3; *p < 0.05 *vs* SB-Ctr; ^#^*p* < 0.05 *vs* SB-SiNPs

Based on available evidence, exosomes have been considered novel communicators of cell-to-cell communication and regulators of health and disease [41]. To further explore the potential communication between lung epithelial and lung fibroblasts, the exosomes in BEAS-2B supernatants were extracted through conventional ultracentrifugation (Fig. 3A). As manifested in TEM images (Fig. 3B), the extracted exosomes were cup-shaped with the double concave disc. The average particle size of exosomes was 118.4 nm in the Ctr group and 122.5 nm in the SiNPs group (Fig. 3C and Table S3). Also, exosomes were identified by Western blot (Fig. 3D), which were positive for CD9, CD63, and TSG101 and negative for calnexin. As demonstrated in Fig. 3E, the DIO-labeled Ctr- or SiNPs-exosomes could be effectively transported into MRC-5 cells. Further, to confirm whether exosomes mediated lung epithelium-fibroblast communication upon SiNPs stimulation, GW4869, an inhibitor of exosome production and release, was applied (Fig. 4A). As manifested in Fig. 4B, GW4869 markedly reversed SiNPs-induced HYP release from MRC-5 cells, indicating that inhibition of exosomes alleviated SiNPs-induced collagen deposition. Consistently, SB-SiNPs exposure triggered activation of MRC-5 cells, while GW4869 could inhibit it, as illustrated by α-SMA immunofluorescence staining (Fig. 4C) and Western blot (Fig. 4D, E). Accordingly, inhibiting exosomes released by GW4869 could significantly alleviate SiNPs-induced fibroblast activation, collagen, and ECM deposition. That is to say, exosomes derived from BEAS-2B served as key mediators involved in the lung epithelium-fibroblast communication upon SiNPs exposure, contributing to fibroblast activation and the aforementioned fibrogenesis in the lungs.

### Exosomal miR-494-3p derived from BEAS-2B mediated TGF-β/BMPR2/Smad pathway via targeting BMPR2 to contribute to fibroblast activation and resultant collagen deposition in the lungs

**Fig. 5:**
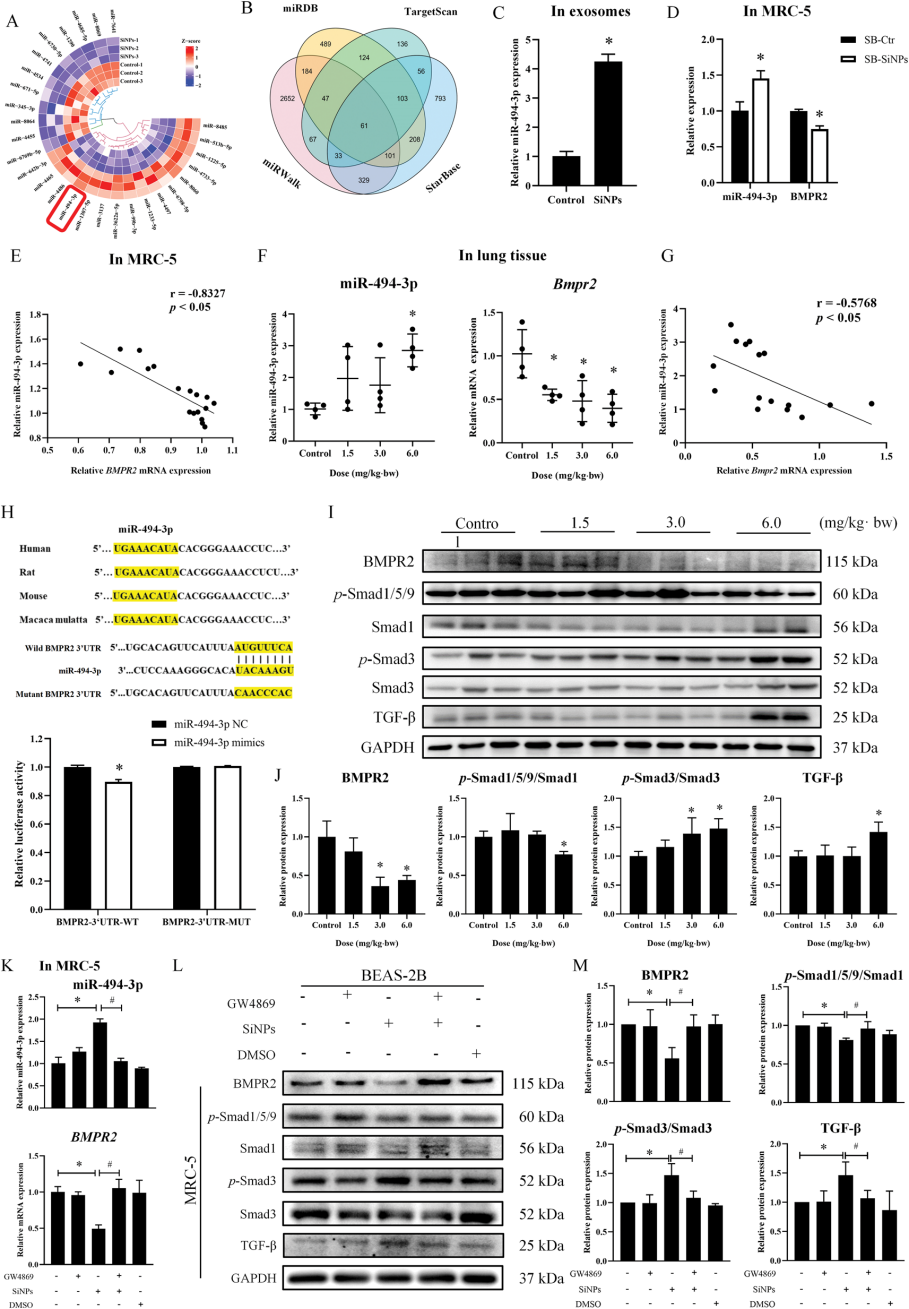
Exosomal miR-494-3p secreted from BEAS-2B cells could target BMPR2 to regulate the TGF-β/BMPR2/Smad pathway in MRC-5 cells. **A** Heatmap of the differential expressions of miRNA in exosomes derived from BEAS-2B. Differential miRNAs with the absolute fold change (FC) > 1.5 and *p* < 0.05 were shown. **B** Potential target genes prediction from 4 databases that could interact with miR-494-3p. The miR-494-3p level was elevated in exosomes (**C)** and MRC-5 (**D**, left). In contrast, the level of *BMPR2* was decreased in MRC-5 (**D**, right). Consistently, the levels of miR-494-3p and *Bmpr2* were detected in lung tissues (**F**). N = 4; *p < 0.05 *vs* control. Meanwhile, the correlation between miR-494-3p and *BMPR2* was conducted in vitro (**E)** and in vivo (**G**). (**H**) Dual luciferase assay verified the targeting relationship between miR-494-3p and *BMPR2*. **I**, **J** The molecular expressions of the TGF-β/BMPR2/Smad signaling pathway in the lung tissues, including BMPR2, Smad1,* p*-Smad1/5/9, Smad3, *p*-Smad3, and TGF-β. N = 3; *p < 0.05 *vs* control. The levels of miR-494-3p and *BMPR2* (**K**), and protein expressions of BMPR2, Smad3,* p*-Smad3, Smad1,* p*-Smad1/5/9 and TGF-β (**L**,** M**) were measured in MRC-5 cells treated with supernatants of BEAS-2B (Ctr, SiNPs, GW4869, GW4869 + SiNPs, or DMSO). N = 3; *p < 0.05 *vs* SB-Ctr; ^#^*p* < 0.05 *vs* SB-SiNPs

To ensure which exosome-linked mediators may trigger fibroblast activation upon SiNPs exposure, we investigated the exosome-packaged abundance of miRNAs (Fig. 5A). We filtered out a highly conserved miR-494-3p, which was increased after SiNPs exposure. The target genes of miR-494-3p were predicted by four databases, i.e., miRWalk, miRDB, TargetScan, and StarBase (Fig. 5B). Among these candidate target genes, BMPR2 is closely associated with lung injury and collagen hyperplasia [42]. In agreement with the microarray analysis, miR-494-3p expression in SiNPs-exosomes (Fig. 5C) and SB-SiNPs-treated MRC-5 cells (Fig. 5D, left) were remarkably elevated. Meanwhile, the expression of BMPR2 declined (Fig. 5D, right) in SB-SiNPs-treated MRC-5 cells, which was inversely correlated with the corresponding miR-494-3p level in fibroblast cells (Fig. 5E). In line with the in vitro results, the miR-494-3p expression was dose-dependently augmented in SiNPs-instilled rat lung tissues, accompanied with a gradually declined *Bmpr2* level (Fig. 5F). A negative correlation was also manifested in Fig. 5G. Meanwhile, the dual-luciferase reporter (Fig. 5H) verified BMPR2 as a target gene of miR-494-3p by binding with the 3'-UTR of BMPR2. BMPR2 is confirmed as a TGF-β family receptor and is deemed to be involved in cell differentiation, phenotype alteration, etc. [43]. Impaired TGF-β/BMPR2/Smad signaling is a crucial contributor to the pathogenesis of pulmonary fibroplasia [44]. Thereby, molecules involved in the TGF-β/BMPR2/Smad pathway were detected in the rat lungs. As manifested in Fig. 5I, SiNPs increased *p*-Smad3 and TGF-β whilst decreasing BMPR2 and *p*-Smad1/5/9 (Fig. 5J), indicating activated TGF-β/Smad signaling but depressed BMPR2/Smad signaling upon SiNPs exposure. In particular, the increased miR-494-3p level in SB-SiNPs-treated fibroblasts could be fully blocked by GW4869 pretreatment in BEAS-2B, moreover, reduced BMPR2 and ensuing disturbed TGF-β/BMPR2/Smad signaling were completely reversed (Fig. 5K–M). Hence, we supposed that the exosomes-packaged abundance of miR-494-3p from lung epithelial cells was a key mediator responsible for fibroblast activation and resultant pulmonary collagen deposition induced by SiNPs, probably mediated through TGF-β/BMPR2/Smad pathway via targeting BMPR2.

**Fig. 6:**
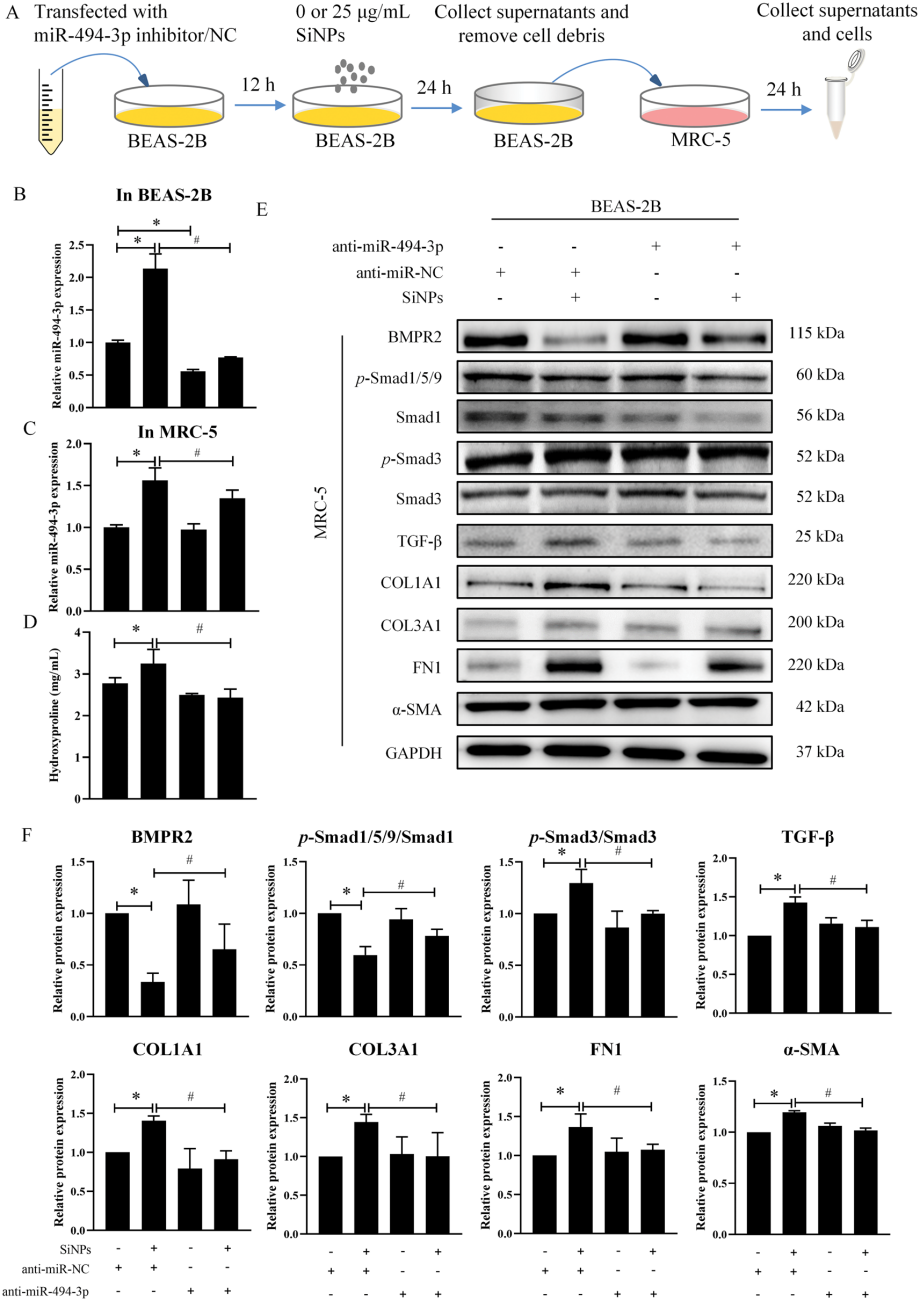
miR-494-3p derived from SiNPs-treated BEAS-2B cells promoted fibroblast activation through the TGF-β/BMPR2/Smad pathway. **A** Experimental flowchart. In brief, BEAS-2B cells were transfected with anti-miR-494-3p or anti-miR-NC combined with SiNPs (25 μg/mL) for 24 h. Subsequently, MRC-5 cells were treated with the corresponding collected and filtered supernatants for 24 h. The expression of miR-494-3p was detected in BEAS-2B cells (**B)** and MRC-5 cells (**C**). **D** The content of collagen in supernatants of MRC-5 was determined. **E**, **F** The expressions of BMPR2, *p*-Smad1/5/9, Smad1, *p*-Smad3, Smad3, TGF-β, COL1A1, COL3A1, FN1 and α-SMA were quantified in MRC-5 cells by Western blot. N = 3; *p < 0.05 *vs* SB-NC-Ctr; ^#^*p* < 0.05 *vs* SB-NC-SiNPs. **SB-NC-Ctr**: After transfected with anti-miR-NC, BEAS-2B cells were treated with DMEM for 24 h, and the supernatants of BEAS-2B were collected. **SB-NC-SiNPs**: After transfected with anti-miR-NC, BEAS-2B cells were subjected to SiNPs for 24 h, and the supernatants of BEAS-2B were collected

**Fig. 7:**
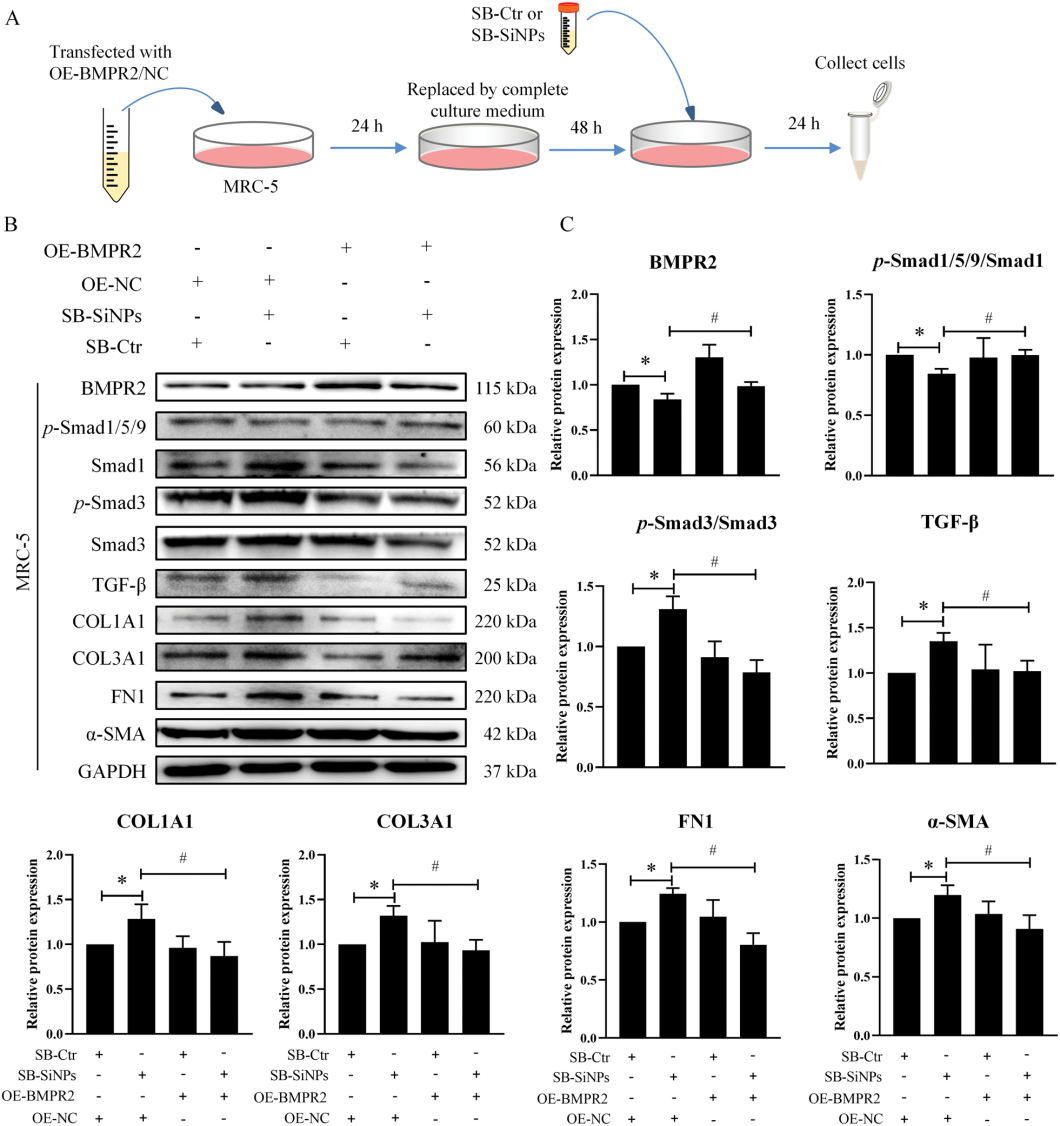
Upregulation of BMPR2 attenuated SiNPs-induced fibroblast activation. **A** Experimental flowchart. Briefly, MRC-5 cells were transfected with lentivirus vectors with BMPR2 or NC for 72 h and then treated with supernatants of BEAS-2B (SB-Ctr or SB-SiNPs) for 24 h. **B**, **C** Relative protein levels of BMPR2, Smad1, *p*-Smad1/5/9, Smad3, *p*-Smad3, TGF-β, COL1A1, COL3A1, FN1 and α-SMA in MRC-5 cells were analyzed. N = 3; *p < 0.05 *vs* OE-NC combined with SB-Ctr group; ^#^*p* < 0.05 *vs* OE-NC combined with SB-SiNPs group

Next, the impact of miR-494-3p on SiNPs-elicited fibroblast activation and collagen deposition was explored by using anti-miR-494-3p (Fig. 6A), which successfully inhibited the miR-494-3p level in BEAS-2B (Fig. 6B) and SB-SiNPs-treated MRC-5 cells (Fig. 6C). The inhibition of miR-494-3p greatly attenuated collagen deposition and rescued the disturbed TGF-β/BMPR2/Smad pathway in MRC-5, as indicated by reduced HYP content (Fig. 6D) and Western blot assay (Fig. 6E and F). Also, whether BMPR2 directly triggered SiNPs-caused respiratory toxicity by mediating TGF-β/BMPR2/Smad pathway was in-depth investigated by interfering BMPR2 in MRC-5 cells using lentivirus vectors with BMPR2 (Fig. 7A). As a result, the overexpression of BMPR2 remarkably suppressed SiNPs-induced disruption of TGF-β/BMPR2/Smad pathway in fibroblasts, resulting in the blockage of fibroblast activation and collagen deposition (Fig. 7B, C). In summary, these manifestations illustrated that miR-494-3p in exosomes secreted from SiNPs-treated BEAS-2B cells targeted BMPR2 to regulate TGF-β/BMPR2/Smad signaling in fibroblasts, leading to fibroblast activation and collagen deposition.

## Discussion

Over the last two decades, significant development has been made in evaluating the health effects caused by SiNPs exposure. Following the respiratory inhalation, SiNPs could directly contact and accumulate in the lung tissues [45], resulting in lung injuries and collagen hyperplasia [46]. However, limited data are available in terms of pathogenesis and molecular mediators accounting for observed pathological lesions. In the present work, pulmonary injury and ECM deposition have successfully occurred upon chronic exposure to SiNPs, and a dose-dependent fibrogenic capability was manifested. Consistent with Yu et al. research [47], we observed that SiNPs induced widened alveolar septa, formation of various cell nodules, and collagen deposition in the rat lungs (Fig. 1B-D), especially at a higher level (6.0 mg/kg·bw). In accordance with our previous publishing [4], the lung impairments elicited by SiNPs were also dependent on exposure of duration, as characterized by alveolar structure destruction and inflammatory infiltration at an early stage, and gradually progressed into fibrotic lesions upon persistent particle stimulation. Moreover, our experimental results showed that in the lung tissues of SiNPs-exposed rats, COL1A1, FN1, COL3A1, and α-SMA were dose-dependent upregulated (Fig. 1G, H), indicating fibroblast activation and collagen deposition to promote the pulmonary fibrogenesis. The dose-response effect of SiNPs on the lung tissues hinted great concern should be paid for exposure control, especially to SiNPs-handling workers. Pitifully, there is no established occupational exposure limit for SiNPs hitherto. In light of quite a slight damage upon SiNPs exposure at the dose of 1.5 mg/kg·bw, our data may provide a valuable reference to policymakers to ensure nano-safety.

The insult to epithelial cell integrity by inhaled toxicants may initiate lung impairments or diseases via the cross-talk between epithelial cells and fibroblasts [48]. When the lung epithelial cells are injured, a process of interstitial repair is triggered through the activation of fibroblasts. However, if this activation is abnormal, leading to excessive proliferation and ECM deposition, that may result in the progression of fibroplasia [49, 50]. Owing to the ample evidence of lung epithelial cell injury caused by SiNPs [51–53], we next investigated the lung epithelium-fibroblast communication in response to SiNPs stimulation. Indeed, the supernatants from SiNPs-stimulated BEAS-2B cells facilitated fibroblast proliferation (Fig. 2B, C), migration (Fig. 2D), and activation (Fig. 2E–G). Nevertheless, the specific molecular mechanisms have not yet been elucidated.

Exosomes are regulators of intercellular communication by transforming their contents, which can be affected by external stimuli [54]. In recent years, exosome-based mechanisms in tissue and organ damage have attracted more and more attention. Enhanced EVs secretion and function were reported in fibrotic patients and animal models [55, 56], where EVs act as carriers for signaling mediators and thus contribute to fibrogenesis. In this study, the inhibition of exosome secretion from lung epithelial cells by GW4869 can greatly alleviate the activation of fibroblasts and ECM deposition by SiNPs (Fig. 4), hinting strategies targeting exosomes may be a potential way against the transformation of pulmonary fibroblasts into myofibroblasts. Consistent with our results (Fig. 4), Yu et al*.* [10] ever presented primary human bronchial epithelial cells-derived EVs mediated myofibroblast differentiation in lung fibroblasts. Likewise, exosomes from hypoxia-caused epithelial cells were verified to promote lung fibroblast proliferation and trans-differentiation, probably by perturbing miR-30d-3p-regulated HSF1 inhibition in a HOTAIRM1-dependent mechanism [57].

In the pulmonary fibrogenic process, epithelial injury triggers the generation of damage-associated molecular patterns (DAMPs), initiating inflammatory pathways and ultimately contributing to the pro-fibrotic response mediated by mesenchymal cells [58]. It is worth noting that exosomes derived from various cell types, including macrophages, epithelial, and fibroblasts, serve as potent mediators of intracellular communication and contribute to the pathological development of lung fibrosis [59, 60]. For instance, exosomes derived from epithelium in lung tissues demonstrated pro-fibrotic effects by inducing pulmonary fibroblast activation [61] and modulating alveolar macrophages by targeting RGS1-mediated calcium signaling, thereby regulating the immune response [62]. Exosomes derived from lung fibroblasts promoted the generation of mitochondrial reactive oxygen species, contributing to mitochondrial damage and resultant senescence in lung epithelial cells [63]. Also, exosomes from macrophages were shown to increase the proliferation of pulmonary interstitial fibroblasts and promote the progression of pulmonary fibrosis [64]. Thereby, exosomes have been considered to be potential biomarkers for diverse lung diseases. Recent studies have shown that the inhibition of exosome secretion by GW4869 has great potential in the treatment of lung injury [65] and silica-induced pulmonary fibrosis [66]. Intriguingly, exosomes also manifested superior therapeutic benefits in lung diseases. For instance, Dinh et al. [67] indicated that inhaled lung spheroid cell exosomes could promote pulmonary repair. Normal human bronchial epithelial cells-derived EVs were reported to inhibit TGF-β-mediated lung fibroblast activation [35].

Exosomes are rich in miRNAs and regulate the biological functions of recipient cells by transporting these miRNAs [68, 69]. Abnormal expression profiles of miRNAs were reported in patients, animals, and cell models of pulmonary disease [70–72]. As manifested in previous studies, exposure to particles resulted in lung injury and fibrogenesis by modulating miR-494-3p, miR-21, and miR-96 [73–75]. Of note, miR-494-3p has been known to mediate multi-disease processes, as manifested by enhancing inflammatory response [76], increasing cell proliferation and migration [77], and cellular senescence [78]. Moreover, EVs derived from lung fibroblasts from patients with pulmonary fibrosis were rich in miR-494-3p, which could accelerate epithelial phenotypic changes [63]. In particular, the exosomal miR-494-3p expression was positively correlated with the disease severity [63]. As our results elicited, miR-494-3p was upregulated in SiNPs-exposed rat lung, BEAS-2B cells, and its exosomes (Fig. 5A-F). Meanwhile, following the inhibition of miR-494-3p in BEAS-2B cells by anti-miR-494-3p, the enhanced expressions of COL1A1, COL3A1, α-SMA, and FN1 in MRC-5 cells by SB-SiNPs were almost completely abolished (Fig. 6). These observations suggested that lung epithelium-derived exosomal miR-494-3p may a feasible mediator for SiNPs-caused pulmonary collagen deposition through promoting fibroblast activation.

miRNAs are known to regulate gene silencing through the post-transcriptional mRNA degradation or repression of the expressed proteins [79]. Here, we illustrated that miR-494-3p was a regulator to target BMPR2 (Fig. 5H) to promote profibrotic phenotypes. Referring to previous studies, BMPR2 was declined in the fibrotic lung tissues of patients and animal models [42, 44, 80, 81] and negatively related to the TGF-β/Smad pathway activation [81, 82]. In our work, BMPR2 and the phosphorylation of Smad1/5/9 were dramatically reduced in SiNPs-exposed rat lungs and fibroblasts, accompanied by elevated TGF-β and ensuing phosphorylated Smad3 (Fig. 5I–M). These data indicated that SiNPs exposure led the TGFβ-BMPR2 balance towards a TGFβ/Smad3 dominant activation direction, resulting in fibroblast activation and accelerating the fibrotic disease development. There is a large body of studies that demonstrated that the imbalanced TGF-β/BMPR2/Smad signaling could lead to pathogenic processes in pulmonary fibroblast activation and collagen deposition [81]. In particular, BMPR2 overexpression could greatly alleviate SiNPs-induced fibroblast activation, which was mediated by the recovery of impaired TGF-β/BMPR2/Smad signaling (Fig. 7). Concertedly, Fukihara et al. [83] indicated that transduced BMPR2 into the lung fibroblasts could suppress phosphorylation of Smad2/3. Switch-independent 3a promoted BMPR2 expression through methylation regulation to inhibit pulmonary arterial smooth muscle cell proliferation [84]. Overall, these findings distinctly demonstrated that BMPR2 depletion is a crucial hallmark of pulmonary fibrotic phenotype.

Here, our findings firstly established the relationship among exosomal miR-494-3p, BMPR2, and TGF-β/Smad pathway in SiNPs-induced lung fibroblast activation and ECM deposition. In detail, SiNPs promoted the miR-494-3p abundance in epithelial exosomes to enhance miR-494-3p in fibroblasts, resulting in imbalanced TGF-β/BMPR2/Smad signaling via targeting BMPR2 and consequent fibroblast activation (Fig. 5). Conversely, the inhibition of miR-494-3p in lung epithelial cells enhanced BMPR2 and *p*-Smad1/5/9 in fibroblasts, and inhibited TGF-β and *p*-Smad3 expressions, leading to weakened fibroblast activation (Fig. 6). In agree with our findings, Zhang et al*.* ever reported elevated miR-494 expression in tumor, which exerted promotive effects on cell proliferation and migration by targeting SIRT3/TGF-β/Smad signaling [85]. By contrast, the downregulation of miR-494 could inhibit TGF-β/Smad signaling from impeding cell proliferation of urethral epithelial cells [86].

Admittedly, this work has some limitations. Apart from epithelial cells, exosomes are released from other lung cells and various immune cells in the lungs. The complexity of the cellular interactions involved in exosome-mediated responses to SiNPs poses a challenge in dissecting the precise mechanisms underlying the observed effects. The use of in vitro models to study exosome-mediated lung injuries induced by SiNPs may not fully capture the dynamic and multifaceted nature of the in vivo environment. Therefore, more relevant cell-culturing models are needed in future studies to further comprehensively confirm the multi-cell communication in the lung microenvironment. Besides, more in vivo work should be carried out to validate the blockade of exosome secretion (e.g., GW4869) or inhibition of exosomal miR-494-3p level, which may be a feasible strategy against lung injury and fibrosis caused by SiNPs.

## Conclusions

In the light of mega-production, extensive application, and increased respiratory exposure of SiNPs, this study aimed to discover the mechanism underlying SiNPs-elicited lung impairment from the perspective of cross-talk between respiratory epithelial injury and fibroblast activation, with a focus on exosomes and its packaged miRNAs. These data firstly expounded that epithelium-derived exosomes promoted pulmonary fibroblast activation and collagen deposition induced by SiNPs via modulating fibrotic signaling pathways and their epigenetic regulations. Specifically, SiNPs exposure could perturb the exosome secretion from lung epithelial cells; in particular, increased exosomal miR-494-3p led to fibroblast activation and collagen deposition through TGF-β/BMPR2/Smad signaling via targeting BMPR2. Overall, our study provided a new perspective on the role of exosomal miRNAs in SiNPs-elicited lung toxicity, as well as a scientific foundation for identifying new targets in the mitigation or treatment for SiNPs-induced lung injury.

### Supplementary Information

Additional file1.

## Data Availability

The datasets used and/or analyzed during the current study are available from the corresponding author upon reasonable request.
