# Supplementary material for: Epithelium-derived exosomes promote silica nanoparticles-induced pulmonary fibroblast activation and collagen deposition via modulating fibrotic signaling pathways and their epigenetic regulations
Source: J Nanobiotechnology. 2024 Jun 12;22:331. doi: 10.1186/s12951-024-02609-y (PMC11170844; doi:10.1186/s12951-024-02609-y)
Supplement: Supplementary file 1 — Additional file1. [file 12951_2024_2609_MOESM1_ESM.docx]

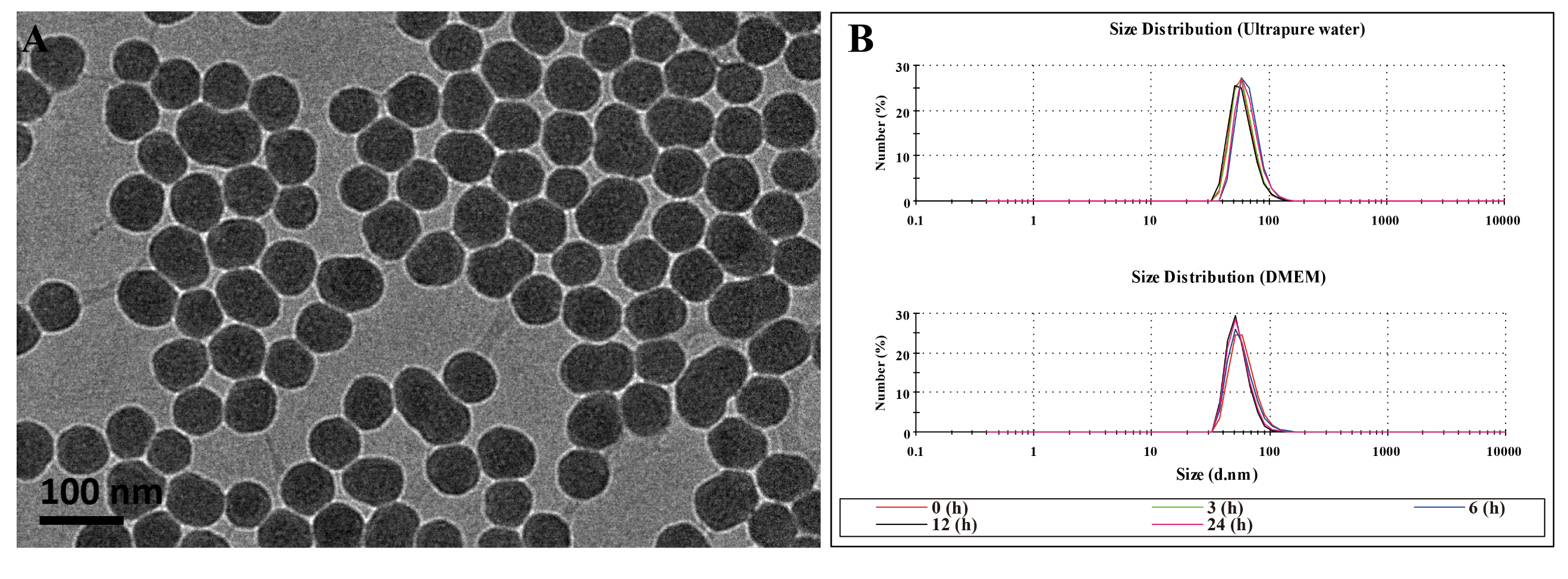


Fig. S1. Particle characterization. (A) Representative TEM image showed the synthesized SiNPs were nearly spherical with relatively well dispersion. The mean particle diameter was about 54 nm as measured by Image J software. (B) The hydrodynamic size of SiNPs in ultrapure water or DMEM within 24 h were measured using a Zetasizer (Malvern Nano ZS90, UK).

**Table S1 Hydrodynamic size and Zeta potential of SiNPs in different media**

| Time (h) | Ultrapure water | | DMEM | | |
| --- | --- | --- | --- | --- | --- |
|  | Hydrodynamic size (nm) | Zeta potential (mV) |  | Hydrodynamic size (nm) | Zeta potential (mV) |
| 0 | 76.21 ± 1.15 | -30.83 ± 1.95 |  | 81.90 ± 1.43 | -23.13 ± 2.37 |
| 3 | 75.56 ± 1.12 | -32.93 ± 0.51 |  | 81.73 ± 1.44 | -24.90 ± 1.31 |
| 6 | 75.82 ± 1.37 | -30.90 ± 3.62 |  | 82.11 ± 1.04 | -22.63 ± 1.23 |
| 12 | 80.46 ± 1.14 | -31.37 ± 2.75 |  | 82.94 ± 0.74 | -22.37 ± 2.15 |
| 24 | 81.49 ± 1.23 | -34.37 ± 2.80 |  | 91.03 ± 0.67 | -21.37 ± 3.67 |

**Table S2. Primer sequences for qPCR**

| Primer names | Sequences |
| --- | --- |
| *Human ACTB* | For: 5’-TGACGTGGACATCCGCAAAG-3’ |
|  | Rev: 5’-CTGGAAGGTGGACAGCGAGG-3’ |
| *Rat Actb* | For: 5’-CCCATCTATGAGGGTTACGC-3’ |
|  | Rev: 5’-TTTAATGTCACGCACGATTTC-3’ |
| *Human COL1A1* | For: 5’-AAAGATGGACTCAACGGTCTC-3’ |
|  | Rev: 5’-CATCGTGAGCCTTCTCTTGAG-3’ |
| *Human COL3A1* | For: 5’-TGAAGGGCAGGGAACAACTTGATG-3’ |
|  | Rev: 5’-GGATGAAGCAGAGCGAGAAGTAGC-3’ |
| *Human FN1* | For: 5’-ACAAGCATGTCTCTCTGCCA-3’ |
|  | Rev: 5’-TCAGGAAACTCCCAGGGTGA-3’ |
| *Human α-SMA* | For: 5’-GTTCCGCTCCTCTCTCCAAC-3’ |
|  | Rev: 5’-ACGCTGGAGGACTTGCTTTT-3’ |
| *Human BMPR2* | For: 5’-CACTCAGTCCACCTCATTCATTT-3 |
|  | Rev: 5’-TTGTTTACGGTCTCCTGTCAAC-3 |
| *Rat Bmpr2* | For: 5’-CACGGACATGCCTTCAGTTT-3 |
|  | Rev: 5’-CAAATGGCAGATGCGAGGAC-3 |
| *miR-494-3p* | 5’-CCGTGAAACATACACGGGAAACCTC-3’ |
| *Anti-miR-494-3p* | 5’-GAGGUUUCCCGUGUAUGUUUCA-3’ |

**Table S3 NTA of exosomes**

| Sample | Diameter (nm) | Concentration (Particles/mL) |
| --- | --- | --- |
| Ctr-exosomes | 118.4 | 2.6*10^7 |
| SiNPs-exosomes | 122.5 | 2.9*10^7 |
